# A Comprehensive Analysis of the Effect of Graphene-Based Dielectric for Sustainable Electric Discharge Machining of Ti-6Al-4V

**DOI:** 10.3390/ma14010023

**Published:** 2020-12-23

**Authors:** Kashif Ishfaq, Muhammad Asad, Saqib Anwar, Catalin I. Pruncu, Mustafa Saleh, Shafiq Ahmad

**Affiliations:** 1 Department of Industrial and Manufacturing Engineering, University of Engineering and Technology, Lahore 548900, Pakistan; kashif.ishfaq@uet.edu.pk (K.I.); 2016im11@student.uet.edu.pk (M.A.); 2 Industrial Engineering Department, College of Engineering, King Saud University, P.O. Box 800, Riyadh 11421, Saudi Arabia; msaleh3@ksu.edu.sa (M.S.); ashafiq@ksu.edu.sa (S.A.); 3 Department of Mechanical Engineering, Imperial College London, Exhibition Road, London SW7 2AZ, UK; 4 Design, Manufacturing & Engineering Management, University of Strathclyde, Glasgow G1 1XJ, UK

**Keywords:** Ti-6Al-4V, graphene, nanoparticles, sustainable machining, material removal rate, tool wear

## Abstract

Titanium alloys, especially Ti-6Al-4V, which is considered a difficult-to-cut material, bears numerous applications in aerospace and biomedical sectors. The criticality of the accurate formation of the machined cavity for the said applications and properties of Ti-6Al-4V accentuated the use of electric discharge machining (EDM). However, the issues of lower material removal rate (MRR) and tool wear (TWR) discouraged the use of EDM. These inherent issues hold a pivotal role regarding the sustainable machining of Ti-alloy. Therefore, in this research the potentiality of kerosene-based dielectric, having graphene nanoparticles, is comprehensively examined for the sustainable EDM of Ti-6Al-4V, which was not focused upon yet. Experimentation was performed under Taguchi’s design (L18) with three types of electrodes, namely Aluminum, Brass and Copper. In total, 36 experiments were conducted, of which 18 were with graphene-mixed dielectric and the remaining were with kerosene. Experimental results reveal that the brass electrode with negative tool polarity yields higher MRR for both types of dielectrics. The maximum MRR (7.602 mm^3^/min) achieved with graphene mixed dielectric is 64.5% greater as compared to that obtained with kerosene (4.621 mm^3^/min). Moreover, the minimum TWR obtained for graphene-based dielectric, i.e., 0.17 mg/min is approximately 1.5 times less than that achieved with kerosene.

## 1. Introduction

Titanium (Ti) and its alloys are extensively employed in the manufacturing of different parts/products in many industrial and commercial applications. Especially, Ti-6Al-4V bears a range of industrial applications, owing to its fabulous characteristics, such as high-temperature stability, remarkable strength-to-weight ratio and excellent corrosion resistance. These attributes make Ti-6Al-4V useful in the chemical industry, medical science, aerospace, automotive industry, energy sector, food industry, and defence industry etc. [1,2,3]. Despite such tremendous properties, Ti-6Al-4V is categorized as a difficult-to-cut material, due to its high chemical reactivity and low electrical and thermal conductivity [4]. The aforementioned factors are a source of notching, chipping and cutting-tool catastrophe. The poor workpiece’s conductivity enhanced the temperature at the tool–workpiece interface, which ultimately reduced the strength of the cutting tool [5]. To moderate the problems that occurred with the cutting of the selected alloy, electric discharge machining (EDM) is considered as a valuable choice. EDM can be employed to cut any conductive material of any strength, hardness and other mechanical attributes [6,7]. Therefore, electric discharge machining is not only employed to manufacture many aerospace, automobile and machine components, but it is also applied for making cavities, intricate shapes in moulds and dies [8]. EDM is also called a “Spark erosion machining process”, in which the erosion from the target surface is realized by the recurring discrete sparking in the spark gap. The tool and the specimen are submerged into a dielectric, which is responsible for providing the stable/controlled spark gap. The flushing of melted debris is also carried out by the available dielectric. It also functions as a heat sink during the cutting of the work part [9].

EDM is considered as a valuable machining technique for the cutting of hard-to-machine materials, such as Ti-6Al-4V [10,11,12]. However, the use of EDM is neglected because of its two intrinsic issues, namely lower material removal rate (MRR) and tool wear. The appreciable level of MRR is essentially required to justify the process economics, whereas the minimum/negligible tool wear rates are the prerequisite for the achievement of a better machine profile’s dimensional accuracy. The aforementioned perspectives have a direct bearing with respect to the sustainable cutting of Ti-6Al-4V [13]. The aspect of minimizing tool wear accounts towards the reduction in material wastage, which is the demand of the sustainable cutting approach. Besides that, the increase in MRR with the same level of input electrical energy is also linked with the sustainable cutting of the selected material. This high MRR at low energy input demonstrates a positive impact on the environment as a lower amount of fuel is used for producing that requisite amount of energy. Thus, this helps to achieve a clean environment [14]. Considering the importance of the issues related to the cutting of the Ti-alloy, both MRR and TWR have been taken as response parameters for this research.

MRR is described as the volume of the material dislodged from the specimen during the machining time. The productivity of EDM typically depends on the MRR, which reflects the operative speed of production [15]. Another important response is tool wear rate (TWR), which has a direct effect on the dimensional accuracy and surface quality of the cut profile/cavity. TWR is described as the weight of the tool material removed in a cutting duration. It has been observed that, if TWR is high, then the inferior quality of the machined surface has been obtained [16]. There are two types of EDM input variables, i.e., electrical and nonelectrical parameters. Parameters such as voltage, current, polarity, pulse time on/off etc. belong to former category. On the other end electrode material, flushing time, and setup time are considered as non-electrical parameters [17]. The selection of EDM variables significantly impacts MRR and TWR. Multiple studies were conducted on electric discharge cutting of the chosen workpart using various electrodes like copper, brass, graphite and aluminium to evaluate their impact in conjunction with other EDM variables on different aspects of surface quality. Chen et al. [18] examined the performance of two types of dielectrics, i.e., kerosene and distilled water on cutting characteristics of Ti-alloy. They proposed MRR and electrode wear ratio is lesser and greater, respectively, if kerosene is used. Moreover, a large number of cracks were found on the surface of workpiece if distilled water is employed as dielectric. Another researcher [19] investigated the performance of machining attributes of Ti-6Al-4V in combination with ultrasonic machining. They studied the effect of different factors, which include type of dielectric, size and concentration of particles, discharge current and pulse on time, on the multiple responses (MRR, EWR, roughness, and depth of redeposited layer). They justified that the blend of both EDM and ultrasonic cutting can upsurge the MRR and hampers the depth of the redeposited layer. In another study, it was cited that MRR and TWR are significantly increased using deionized water as a dielectric in the EDM of Ti-alloy [20]. Pradhan et al. [21] stated that on-time was the main influencing factor for MRR and overcut in EDM of the selected workpiece, whereas current was most significant factor for TWR in the micro-EDM of Ti-6Al-4V. In another work, conducted on the same material, it was reported that the electrode and its polarity determine recast layer formation [22]. Tariq et al. [23] examined the role of four types of dielectric fluids in relation to MRR with two types of electrodes at positive and negative polarities. It was claimed that better machining of the low carbon steel workpiece was achieved by tap water. In the EDM of TiB2, where the Cu electrode is used, it was stated that the current was the prime influencing variable for MRR, whereas TWR on-time was ranked as the leading control parameter [24]. The cutting performance comparison of two dielectrics, i.e., kerosene oil and distilled water, was done in terms of MRR and TWR in the EDM of Ti6Al4V. The cutting performance of distilled water was superior in comparison to kerosene oil [18]. Muthuramalingam [25] investigated the effect of a dielectric, which is a blend of tap and deionized water for the electric discharge cutting of Ti6Al4V. The cutting potential of the dielectric was ranked based on the MRR obtained during the EDM of Ti-alloy. It was observed that MRR is primarily dependent on the conductivity of the dielectric.

Many researchers have revealed that the dielectric with particles is generally preferred for improving MRR and TWR in EDM. Erden and Bilgin [26] investigated the use of powders of Cu, iron, carbon and aluminium with the dielectric in electric discharge machining. It was suggested that rate of machining was increased at a high concentration of powder. In the same way, Jeswani [27] experimentally confirmed that MRR was enhanced by about 60% and TWR was condensed at around 15% when graphite powder was added in kerosene in a concentration of 4 g/L in the EDM process. Wong et al. [28] and Yan et al. [29] reported that the various characteristics of cut-surface, such as hardness, wear and corrosion resistance, were notably enhanced by adding nano-powder in the dielectric. The previous study in this area shows that the debris’ availability affects the strength magnitude of the electric field. It means that there is a relationship between electric field’s strength and the concentration of debris. Murray et al. [30] observed that if low off-time was used then all debris are not flushed which not only lessen the material erosion but also led to short circuiting. Thus, the presence of conducting particles in the dielectric medium helps in the effective evacuation of debris that could result in increased gap size, which ultimately yields greater MRR and high surface quality. Basically, the particles’ availability in the dielectric changes/adjusts the spark gap, according to the size of the particles used. Such an adjustment of spark gap helps in the efficient removal of melted debris and, subsequently, better surface finish was obtained, as compared to that achieved with pure dielectric [31,32,33]. For instance, the influence of Ti powder-based dielectric was investigated in terms of surface integrity in the EDM of D2 steel. The results revealed that the application of Ti nano-powder in the dielectric yields better MRR and surface finish [34]. In another work, it was reported that Ti nanoparticles also improve the micro-hardness of the layer redeposited at the machined profile [35]. Similar findings regarding the increment in the surface hardness were also noted when molybdenum particles were used in the dielectric [36]. The addition of particles even results in the improvement in surface properties, such as hardness, water repellency and Vickers hardness as well. Further, the addition of chromium particles in the dielectric provides a corrosion-resistant layer on the cut cavity during the EDM of alloy steel SKD11 using a copper electrode [37]. The inclusion of Al_2_O_3_ nanoparticles in deionized water lowers the micro-cracks on the machined cavity in the EDM of super alloy Inconel 825 with a copper electrode [38]. Another important attribute that determines the efficacy of a powdered mixed dielectric in EDM is its concentration. It was narrated that the magnitude of TWR reduces with a rise in MRR, as the concentration of boron carbide particles is increased during EDM of Ti-6Al-4V. The research determined that TWR is increased when particles are added up to 5 g/L, but an opposite trend was noticed when the concentration was raised further [39]. The outcome of the rise in concentration of graphite powder in the dielectric in EDM of Ti6Al4V was reported in another study. Initially, MRR was increased as the concentration of powder was upsurged and afterwards the enhancement of powder concentration yields lower MRR. It was explained that the accumulation of powder in the spark gap condenses the transitivity and, thus, the MRR achieved was compromised [40].

Although significant efforts have been made in the past for achieving high MRR and low TWR in the EDM of Ti6Al4V, considering different means, i.e., by adding certain additives or by adjusting the parameters or by some modification in the cutting mechanism. However, the potential of graphene nanoparticles mixed with kerosene dielectric has not been explicitly studied so far in the EDM of Ti6Al4V. It is pertinent to mention that graphene has attracted the focus of the researchers, owing to its remarkable characteristics. Therefore, this research aims to comprehensively inspect the influence/impact of graphene-based dielectric on the EDM of Ti6Al4V in terms of MRR and TWR. The selection of these two response attributes is based on their well-proven contribution towards ensuring the sustainability of the machining process. The achievement of higher MRR by the addition of graphene in the kerosene at same electrical input is a depiction that lesser resources are consumed at the power generation station. So, the low level of pollution contributed to the environment if graphene-based dielectric is engaged in EDM. In other words, it helps to maintain a clean environment. The aspect of TWR is linked with sustainability as it accounts for the wastage of material. Hence, its lower value is deemed necessary for warranting the sustainability of the EDM. Considering the importance of the set responses, experimentation has been planned under Taguchi’s design (L18). In total, 36 experiments were performed: 18 with kerosene and the remaining 18 with graphene-mixed kerosene. The experimental results were analysed with the statistical tools. An optical microscopic analysis has also been done for providing the insight of the parametric effects in relation to the selected responses. Optimal settings have also been developed, based on the parametric trends.

## 2. Materials and Methods

**Figure 1:**
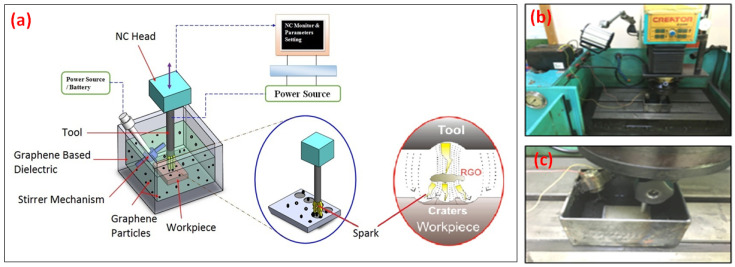
Setup of EDM Die Sinking: (**a**) 3D drawing of experimental setup; (**b**) actual machining setup; (**c**) workpiece setting in the container.

Ti-6Al-4V was taken as a workpiece herein, which has numerous applications in aerospace, automobile, chemical, and biomedical applications [41]. The workpiece used in this study have dimensions 100 mm length, 70 mm breadth, and 20 mm thickness. The composition of the Ti-6Al-4V was evaluated via optical spectrometry and tabulated in Table 1. The salient attributes of Ti-6Al-4V, as taken from reliable literature, are presented in Table 2 [12]. The experiments were performed on an EDM die-sinking machine (model: RJ-230). The experimental setup was adjusted for conducting the experiments with and without powder mixed dielectric. The schematic of the experimental setup used for EDM of Ti6Al4V is presented in Figure 1.

**Figure 2:**
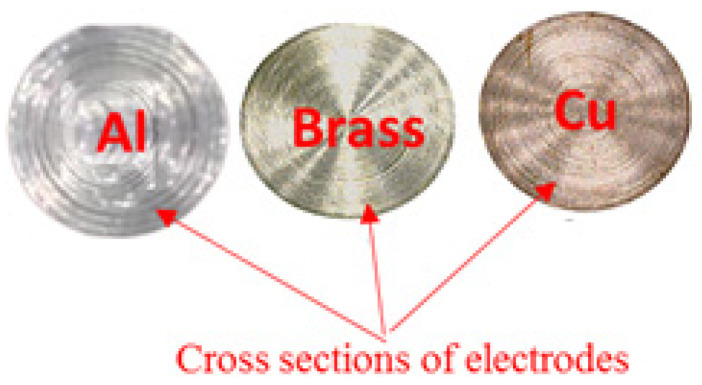
Cross section view of aluminium, brass and copper electrodes.

In this setup, a separate container with a stirrer inside was used for adding graphene nanoparticles in the kerosene. The stirrer was used to ensure the homogeneity of the mixture during experiments. In each of the experiments, a cavity with a diameter of 9 mm and depth of 0.3 mm was produced, using an electrode of 9 mm. The three different electrode materials, namely copper, brass and aluminium were engaged in this research to select the best tool material for the sustainable cutting of the selected work part. The cross sections of all the electrodes are shown in Figure 2. Six control variables, i.e., electrode materials, tool polarity, servo voltage, flushing time, pulse–time ratio and current have been used in this research.

**Figure 3:**
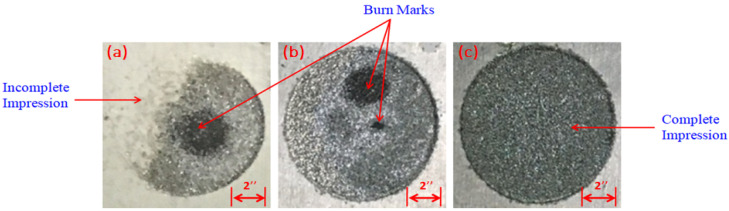
Undesired scenarios of preliminary trial experiments: (**a**) incomplete impression with burn mark; (**b**) burn Marks; (**c**) complete impression.

Pulse time ratio (PTR) is the ratio of on-time to off-time. The selection of these control variables was made because their impact was rated significant with regard to MRR and TWR in the EDM of Ti6Al4V [20,42,43,44]. Preliminary trials were initially conducted to seek the appropriate parametric levels. It has been noticed during initial trials that, at certain parametric values, an incomplete machined impression was realized on the cut cavity, especially at higher values of current and PTR. These undesired scenarios are illustrated in Figure 3a,b. Only those levels were selected, which ensures the provision of complete machining impression, as depicted in Figure 3c. Trials were performed for both types of dielectrics, i.e., kerosene and graphene mixed with kerosene. After careful examination of the effects of the input variables for MRR and TWR using said dielectrics, the parametric levels were defined. Those levels were iterated for mature experimentation, where proper machined cavity can be achieved without any kinds of burn marks. It is pertinent to mention that the same parametric levels were identified on the basis of preliminary trials in order to have the same baseline for the sake of comparison. The factors other than control variables were set as constant. The complete description of parametric levels is tabulated in Table 3.

**Figure 4:**
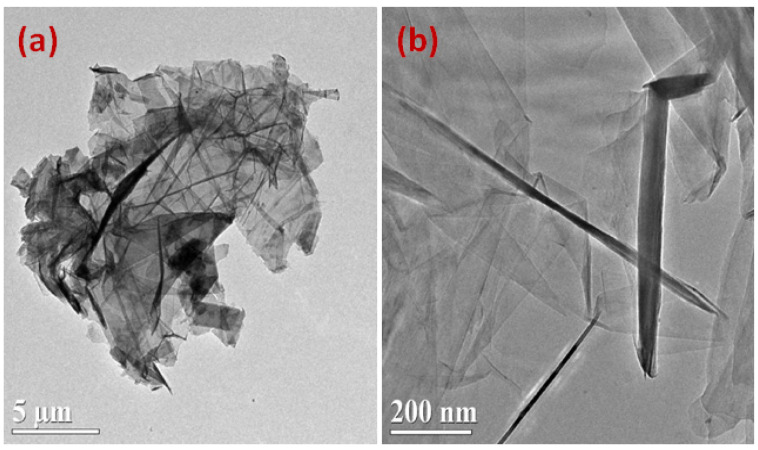
TEM images of graphene nanoparticles at different magnifications; (**a**) at lower magnification, and (**b**) at higher magnification [45].

Experimentation was planned according to Taguchi’s experimental design, which was proved as a promising design technique in a similar study conducted on the same material [13]. Considering the variables selected and their iterated level values, L18 orthogonal design was chosen for performing the experimental trials. In total, 36 experiments were performed, 18 with each type of dielectric (kerosene and graphene mixed with kerosene). The experiments proceeded with graphene-mixed dielectric (size of graphene nanoparticles: 2–10 nm) with a concentration of 0.5 g/L. The salient characteristics of the graphene particles and their morphology are described in Table 4 and Figure 4, respectively [45]. The information of particles provided here is taken from the supplier/manufacturer of the graphene particles, the reference of which has also been cited. Before starting every experiment, the flatness of workpiece and the tool was ensured. In each of the experimental trials, the time of cut was sensibly calculated using a stopwatch. As MRR and TWR are the selected responses, herein, therefore, the masses of the workpiece and the tool were measured before and after each experiment on an electrical balance. 

Initial and final masses of workpiece and tool have been measured. MRR was calculated by volumetric method, whereas TWR was assessed in terms of mass reduction. MRR and TWR were found using the relations mentioned as Equation (1) and Equation (2), respectively.

(1) $$MRR=\;\frac{x\;\times y\;\times d}{t}$$

(2) $$TWR=\;\frac{Mi-Mf}{t}$$

Here, *x*, *y*, *d* and *t* represent lateral dimension, axial dimension, depth and cutting time, whereas, *M_i_* and *M_f_* denote the initial and final masses of the electrode, respectively.

After calculating the MRR and TWR for all the experiments, the results have been comprehensively analysed using statistical techniques, such as parametric line plots and bar charts, etc. These plots have been drawn considering the mean values of the responses. Against each of the parametric levels, the values of MRR have been noted for all the experiments conducted on the specified level value of the parameter. As polarity has two levels, this means that nine experiments were performed at each tool polarity. However, in a case when a parameter has three levels, as in the case of current against each of the levels, six experiments were performed. For analysis purposes, the mean value of the response against the defined parametric level was computed. Since current has three levels, this means three data points are available that are linked with a line to depict a parametric trend for the set response, i.e., MRR. The similar procedure is opted for in the case of TWR. Moreover, optical microscopic analysis was performed to have physical evidence of the findings. A detailed comparison has been made for the results achieved with the graphene-mixed dielectric and simple kerosene. Finally, an optimal combination of input variables has been developed based on parametric analysis.

## 3. Results and Discussion

**Figure 5:**
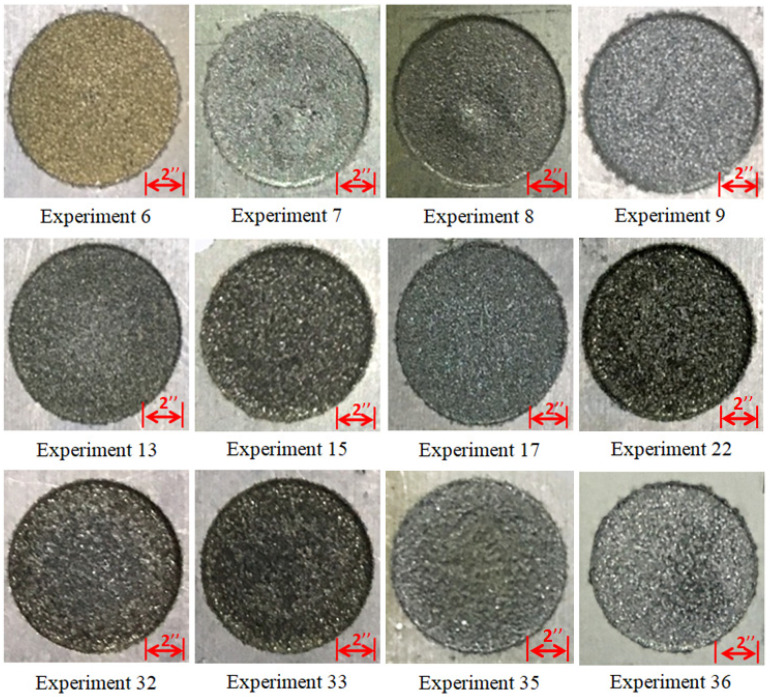
Machined surface of selected experimental trials.

Experimental results are provided in Table 5. A range of values of MRR and TWR has been achieved with different input parameters using three different electrodes. The cut cavities of the selected experimental trials are presented in Figure 5. The detailed elaboration of parametric trends is provided in the forthcoming sections.

### 3.1. Effect of Control Variables on Material Removal Rate (MRR)

#### 3.1.1. Discharge Current

**Figure 6:**
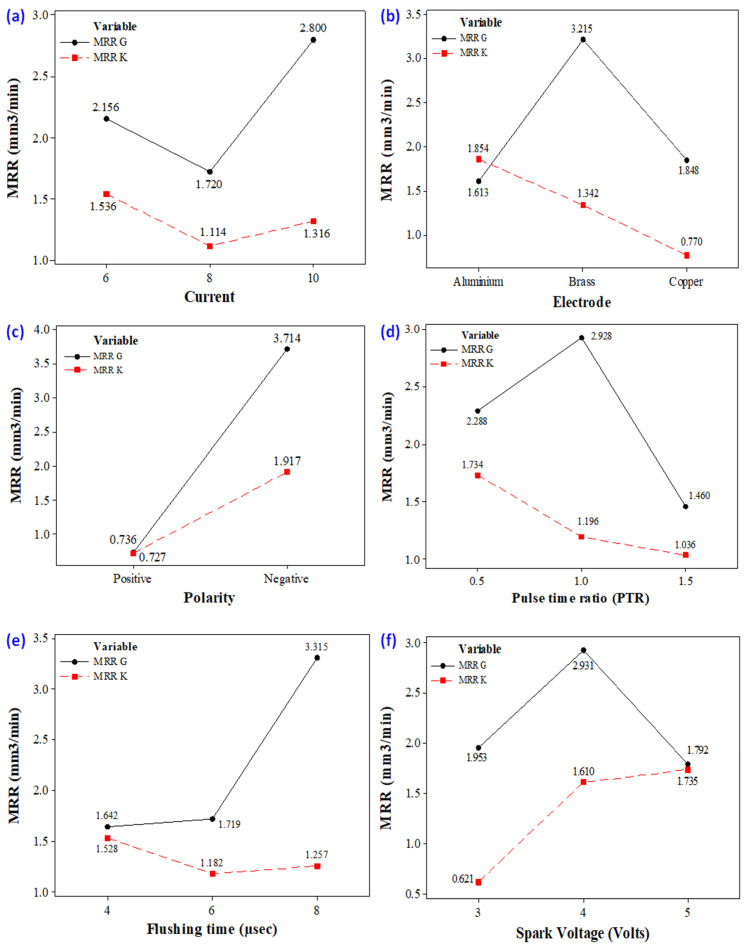
Parametric plots (**a**) discharge current vs. MRR (**b**) electrode vs. MRR (**c**) polarity vs. MRR (**d**) pulse–time ratio vs. MRR (**e**) flushing time vs. MRR (**f**) spark voltage vs. MRR.

It is observed that discharge current is the main governing factor that affects the MRR in the EDM of Ti6Al4V using a graphene-based dielectric, as illustrated in Figure 6a.

**Figure 7:**
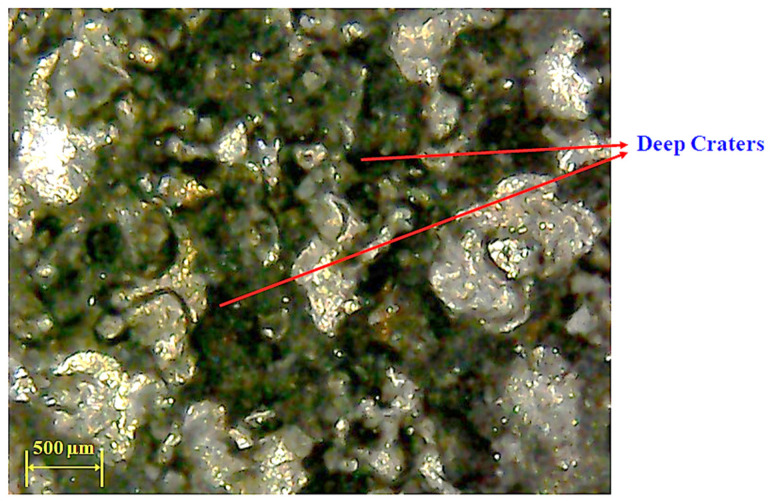
Micrograph of machine cavity showing deep craters at 10 amps using brass electrode.

As the value of current increases from 6 to 8 amps, the MRR decreases because of the presence of graphene particles. Their availability in the discharge gap creates more hindrance in front of sparks, which results in a lesser MRR. However, the magnitude of MRR sharply improved as the discharge current value raised from 8 to 10 amps. The rise in MRR is associated with the generation of intense heat energy in the gap between the tool and workpiece. At high energy, a larger quantity of material was removed in the form of melted debris from the workpiece surface, resulting in higher MRR. In any case, given the large amount of heat input, the higher the magnitude of current, which also yields powerful discharges. These discrete penetrating discharges intensively hit the liquid material in the liquid puddle and caused the ejection of more material, leaving deeper craters in the machined region [46]. The availability of deep craters at a high value of current is evident in the micrographs of machined cavity presented in Figure 7. The subterranean craters’ formation is a proof of larger material removal at a current of 10 amps because of the greater strength of the spark energy, as compared to other levels of current.

The experiment results declared that the kerosene-based dielectric showed a somehow similar trend for MRR, as that was portrayed by the graphene mixed dielectric. The MRR is decreased with the rise in current up to 8 amps due to the deposition of the carbon layer over the surface of the tool, which causes improper discharging between the workpiece and the tool [47]. Subsequently, low MRR is achieved as demonstrated in Figure 6a. Beyond 8 amps, the MRR magnitude is improved because of the higher strength of discharge energy. Thus, for kerosene oil-based dielectric liquid, a greater MRR is achieved at a lower value of discharge current, compared to its other levels, i.e., 8 and 10 amps.

By examining the results (one with a graphene-based dielectric and other with a kerosene-based dielectric), it has been confirmed that the material removal rate is higher for a graphene-based dielectric. This is attributed to the fact that the presence of graphene nanoparticles influences the spark erosion phenomenon in EDM. Their existence in the dielectric fluid provides a powerful explosion, which induces a strong penetrating heat flux in the workpiece. Thus, a higher MRR is achieved in the EDM of Ti6Al4V with the application of graphene nanoparticles in the dielectric in contrast to simple kerosene, as demonstrated in Figure 6a. The line plots of discharge current are clearly depicting that values of MRR are greater for graphene-mixed slurry, as compared to kerosene. Therefore, graphene particles have an important role in order to improve the machining characteristics.

#### 3.1.2. Electrode

**Figure 8:**
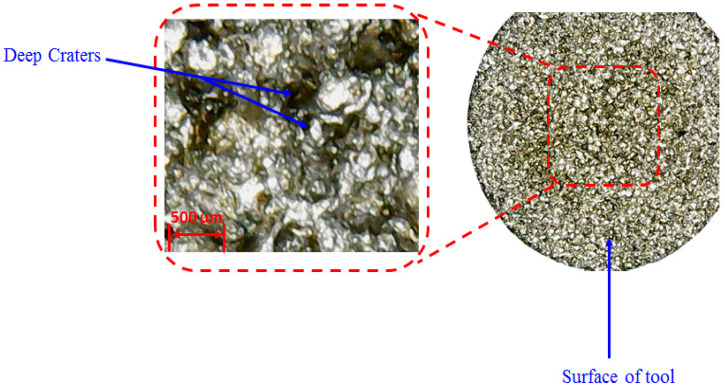
Micrograph of Aluminium electrode.

Three electrodes—aluminium, brass and copper—were used for experimentation. Their effect is shown in Figure 6b. It has been shown that the Brass tool provides the maximum MRR, as compared to MRR, calculated for the other two tool materials when graphene particles are present in the dielectric medium. The primary reason for the high value of MRR is the low thermal conductivity and nonmagnetic property of brass [23]. The graphene particles, which are charged while sparking, did not attach with the tool surface, which produces a smooth electric spark, and the rate of material erosion is higher. On the contrary, in the case of the Cu electrode, the surface of the electrode is shielded with the carbon particles, which create a hindrance in sparking and low MRR is achieved. The minimum MRR was obtained with the use of the Al electrode, while the EDM of Ti6Al4V was obtained using the graphene-based dielectric. The lower melting point of aluminium (660 °C/1220 °F), compared to other electrodes, is the reason behind this shift. During the process of the EDM of the selected material, the electrode (Al) starts wearing more rapidly due to an intense heat flux in the cutting regime in contrast to the workpiece whose melting temperature is significantly higher (1660 °C), as shown the Figure 8.

**Figure 9:**
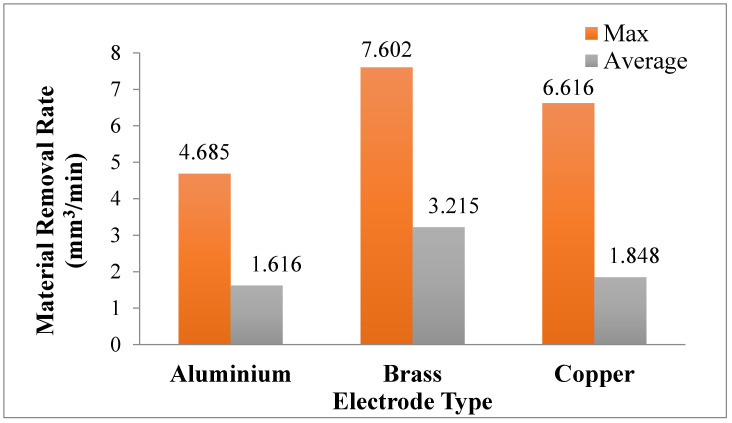
Comparison of three electrodes with MRR in graphene-mixed dielectric.

The cutting performance of all the electrodes was also compared in terms of MRR in the EDM of the Ti-alloy using a graphene-mixed dielectric, as shown in Figure 9. The cutting performance of the brass electrode is appreciable, as compared to the rest of the electrode, when nano-graphene particles are mixed with kerosene. In Figure 9, the maximum and average values of MRR represent the highest and mean values of MRR obtained for both types of dielectrics during the whole experimentation.

**Figure 10:**
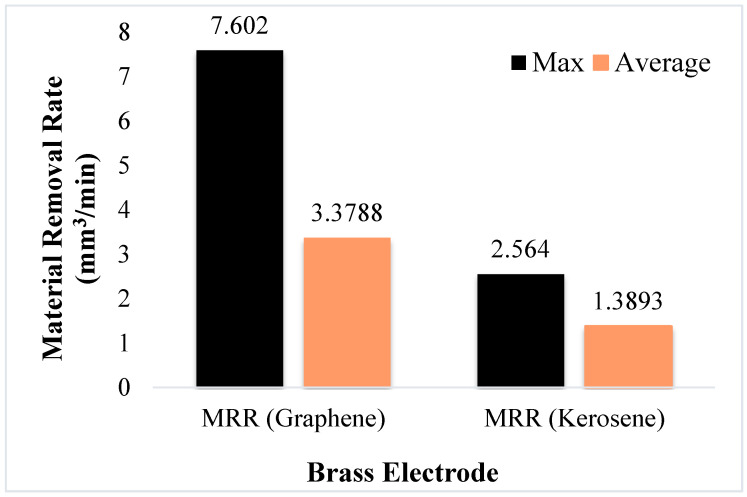
Comparison of MRR obtained in graphene-mixed and kerosene dielectric for brass electrode.

In case of kerosene dielectric, the electrode of Al provides the highest MRR amongst the available alternatives. The decreasing trend of MRR is observed for the kerosene-based dielectric from aluminium to copper, as presented in Figure 6b. The copper electrode provides the lowest value of MRR in comparison to the other two tool materials. The carbon particles during machining were deposited on the surface of tool and decrease the spark capacity which lessened the material erosion on the workpiece surface. As a result, MRR got reduced. Hence, during the EDM of Ti6Al4V, employing kerosene as a dielectric, the maximum MRR can be obtained with the Al electrode. However, this maximum value of MRR (obtained with Al under the kerosene dielectric) is significantly lower when compared with the highest value of MRR, achieved with the brass electrode under a graphene-based dielectric. The results attained with the brass electrode in the two scenarios, i.e., one with kerosene and other with the graphene-based dielectric, are also presented in Figure 10 for the sake of comparison. The MRR provided in the latter case is exceedingly better, as shown in Figure 10. In Figure 10, the maximum value represents the highest MRR value achieved during the experiments, conducted with the brass electrode under both types of dielectrics, whereas the average value of MRR accounts for the mean value of MRR obtained during the experimental trials performed using a brass electrode with kerosene and graphene-mixed dielectrics.

#### 3.1.3. Polarity

**Figure 11:**
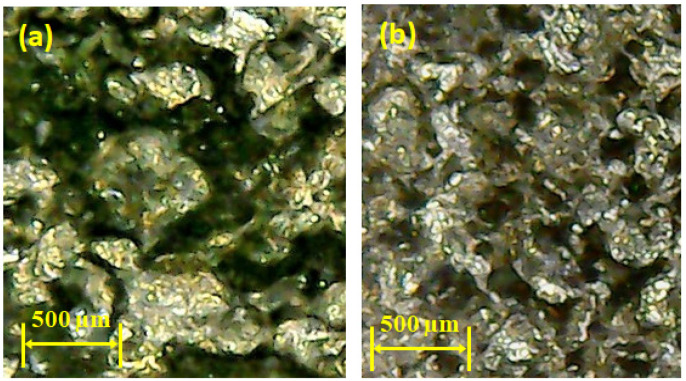
Micrographs representing the effect of polarity on MRR. (**a**) Eroded surface at negative polarity. (**b**) Eroded surface at positive polarity.

Polarity is another important control variable for getting higher MRR. Two types of polarities are engaged in the EDM process, i.e., positive and negative polarity. Usually, polarity refers to the “polarity of the tool”. Positive polarity means positive polarity of the tool and, similarly, negative polarity designates the negative polarity of the tool. In the EDM process, if the tool is positively charged then obviously the workpiece is negative and vice versa. The effect of polarity on MRR in the EDM of Ti6Al4V using both kerosene and graphene-based dielectrics is demonstrated in Figure 6c. The positive tool polarity gives lower MRR for the graphene-based dielectric as well as for kerosene. An almost-similar value of MRR is realized with the chosen dielectrics. Contrarily, at the negative polarity, MRR is increased in both cases. It has been reported that, in the case of negative polarity, more energy is applied at the workpiece than the tool, which erodes the material surface more effectively as compared to energy generated at positive polarity; the same results are also presented in Figure 11 [48].

However, the MRR found at negative polarity with the graphene–kerosene mixture is notably higher in contest to that achieved with kerosene. Hence, negative polarity is more suited when graphene particles are mixed in the dielectric for getting the high value of MRR. It has been confirmed from the results that MRR is higher when machining has been performed at negative polarity because the spark formation is greater, which forced the material to wear out.

#### 3.1.4. Pulse–Time Ratio

**Figure 12:**
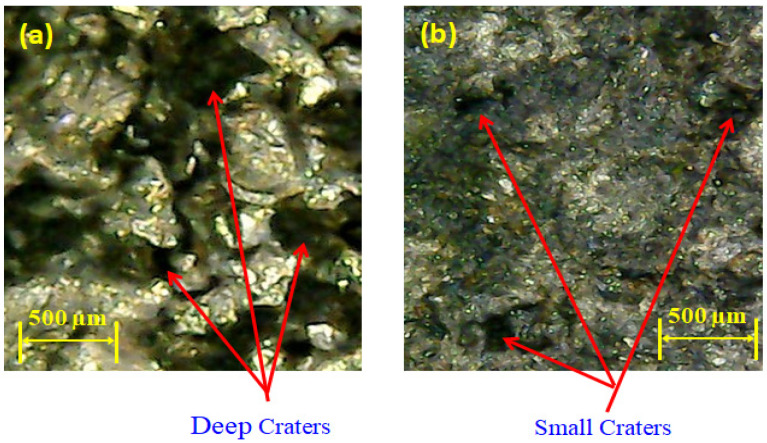
Micrographs showing craters for graphene mixed dielectric: (**a**) deep craters at pulse–time ratio 1.0; (**b**) small craters at pulse–time ratio 1.5.

Pulse–time ratio (PTR) is another important factor which greatly affects the MRR in EDM, as highlighted in Figure 6d. The effect of said parameter is more pronounced in the case of the graphene-based dielectric, as shown in Figure 6d. The pulse–time ratio is the ratio of pulse on-time to pulse off-time. Three different pulse–time ratios (0.5, 1.0 and 1.5) were selected to evaluate their influence on material removal rate. When the pulse on time was upsurged from 25 to 50 µs, while keeping the pulse off time constant at 50 µs, the MRR was improved. The increase in the pulse on-time ensures the availability of discharge energy for longer period in the cutting regime. Subsequently, more heat energy is liberated in the spark gap that led to melt a greater amount of target material. Such a material removal creates deep craters, which are also shown at the machine, as depicted in Figure 12a. However, the rise in pulse on-time beyond 50 µs portrays an opposite trend for MRR, i.e., up to 75 µs MRR is decreased. This reduction is associated to the arcing and deposition of the carbon layer on the surface of the workpiece. In EDM, arcing is a phenomenon in which discharge energy disperses due to the long pulse duration and, consequently, the discharge density within the discharge spot decreases [49]. Thus, small craters are formed at the machined surface that lowers MRR, as demonstrated in Figure 12b. This carbon layer acts as a guard to the spark formation, and ultimately, MRR is compromised.

In the case of the kerosene dielectric, a decreasing trend of MRR is observed for a pulse–time ratio from 0.5 to 1.5, as presented in Figure 6d. This happens because, at higher pulse–time ratio, the plasma channel expands, which causes the negligence in density of discharge energy [50]. Hence, MRR declines.

#### 3.1.5. Flushing Time

Flushing is a phenomenon of removing debris from the gap between tool and workpiece. It is considered as an important function in Electric Discharge Machining. There are various types of flushing techniques, such as pressure flushing, suction flushing, and jet flushing, etc. Each technique has its own application, as per the need and requirement. M.M. Makenzi et al. [51] reported that the proper selection of the flushing method has a significant impact on EDM performance. Furthermore, they proposed that the most preferable flushing technique to optimize the performance parameters is through utilizing the magnetic field in the finishing regime. This would result in higher MRR and better surface finish. Schumacher [52] claimed that a greater amount of debris in the discharge gap generates a continuous arc and/or short circuit, and may affect the stability of the process. In EDM, the dielectric fluid is distributed throughout the surface of the workpiece to remove solid and gaseous debris generated during the operational state and to achieve the optimum temperature of the dielectric well before its flash point. The impact of this control variable for MRR in the EDM of Ti6Al4V under kerosene- and graphene-based dielectrics is illustrated in Figure 6e.

**Figure 13:**
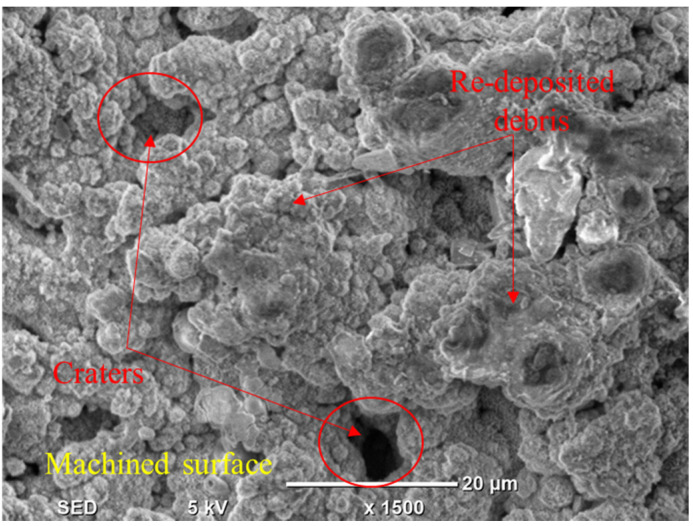
Debris and craters are present on workpiece surface at flushing time equal to 4 µse.

The larger value of flushing pressure provides better MRR when the graphene-based dielectric is employed, as depicted in the Figure 6e. Since flushing time is accountable for the effective removal of melted debris from the cutting zone, so its high value warrants the effective removal of debris. MRR magnitude is lower at 4 µsec because some debris remains on the surface of the workpiece, as presented in Figure 13, and cause hindrance during the material erosion. However, MRR is remarkably amplified when flushing time is enhanced from 4 to 8 µsec. At 8 µsec, the debris are effectively removed from the machining regime. Thereof the chances of re-deposition of the debris are minimized. Hence, MRR is improved. On the other hand, MRR is found to decrease for the other dielectric, i.e., kerosene oil from 4 to 6 µsec because of the quenching of molten metal over the surface of the workpiece. The debris is stacked over the surface of the workpiece instead of being removed. Therefore, a smaller value of MRR is achieved to get reduced. Onward from 6 µsec, the curve slightly increases which results in a higher MRR due to there being no quenching effect and the staking of debris over the metal surface, which increases the chance of debris removal.

#### 3.1.6. Spark Voltage

The effect of spark voltage on the defined response by applying the two types of dielectrics in EDM of Ti-alloy is provided in Figure 6. The trend of spark voltage for MRR is found to be different for both of the dielectrics. For the case of the graphene-mixed dielectric, a high MRR is obtained as the spark voltages upsurge from 3 to 4V, due to greater discharge energy per spark. This provokes the ionization phenomenon between the tool and workpiece. An intense sparking in the cutting region is its outcome, which leads to the removal of more workpiece material. Interestingly, MRR decreases sharply as spark voltage is raised from 4 to 5V. Though at larger values of voltage more discharge energy per spark is generated, if the magnitude of this energy is beyond a certain threshold, it can cause unfavourable breakdown of the dielectric and debris present in the spark gap [53]. Consequently, MRR is lessened. However, for kerosene, the MRR is constantly improved with the rise in spark voltage, as highlighted in Figure 6f. At 3V, lesser spark energy is engendered, and accordingly a smaller amount of material is removed. Contrarily, at 5V, MRR is better, owing to the superior spark strength, which is responsible for the excessive erosion of the material.

### 3.2. Effect of Control Variables on Tool Wear Rate (TWR)

#### 3.2.1. Discharge Current

**Figure 14:**
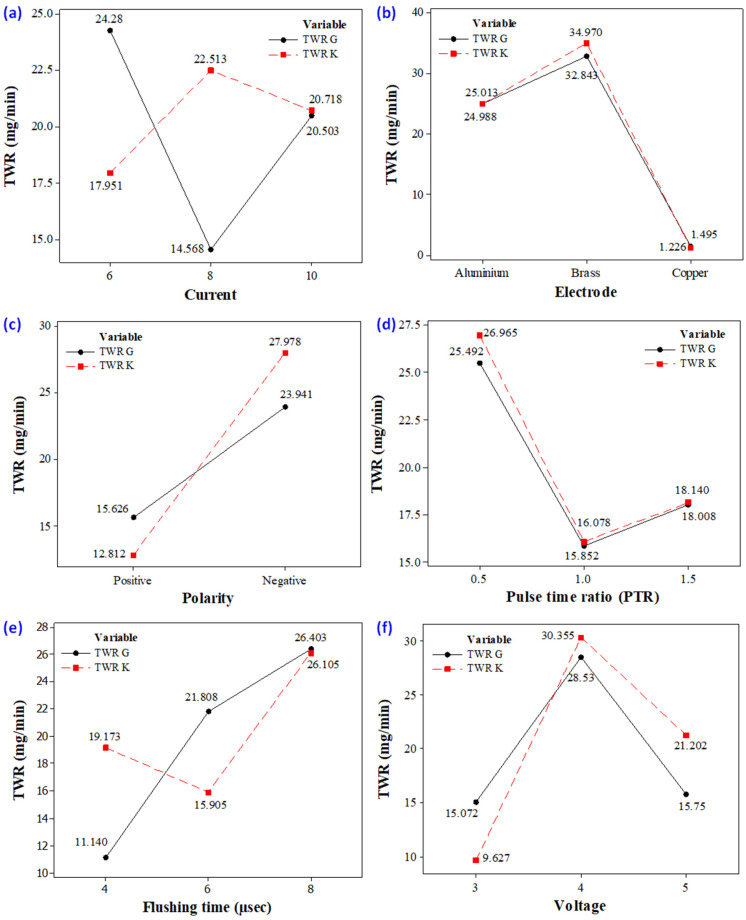
Parametric plots (**a**) discharge current vs. TWR (**b**) electrode vs. TWR (**c**) polarity vs. TWR (**d**) pulse–time ratio vs. TWR (**e**) flushing time vs. TWR (**f**) spark voltage vs. TWR.

**Figure 15:**
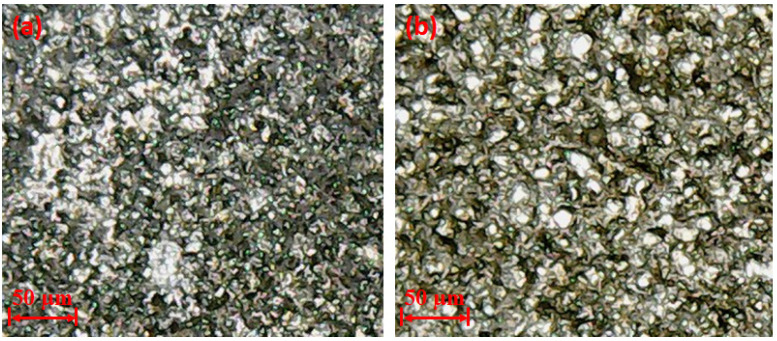
Micrographs representing electrode wear rate at 8A: (**a**) surface of copper tool subjected to less wear in graphene-mixed dielectric; (**b**) surface of aluminium electrode exposed to high wear in kerosene dielectric.

The effect of discharge current on TWR is shown in Figure 14a using the selected types of dielectrics, i.e., graphene-based and kerosene-based. For the case of the graphene-mixed dielectric, TWR declines, initially up to a current of 8 A, and further rises in current, showed by an inverse behaviour with respect to the wearing of the tool, as depicted in Figure 15a. The reason for the reduction in TWR from 6 to 8 A is attributed to the re-melting of the nanoparticles over the surface of the tool again, which acts as a shield against the electric sparking [54]. So, the impact of heat flux for removing the material from the electrode was decreased. On the other end, more wearing of the tool is noted as the current is raised from 8 to 10 A. The larger current value is responsible for providing high discharge energy in the spark gap, which induces strong heat energy to the workpiece surface and the tool as well. This causes the melting of the tool itself and the tool surface is worn out. Keeping in view the above discussion, it is concluded that the 8A current gives better results in terms of TWR in the EDM of Ti-alloy if a graphene-based dielectric is employed.

The micrograph shown in Figure 15b also demonstrates that the surface of the tool is subjected to high wear when the current is enhanced up to 8 A if kerosene is used in EDM. The discharge energy available in the electrode–workpiece gap at a second level of current is greater in contrast to that generated at 6 A, shown in Figure 14a. Therefore, more erosion of material takes place from the electrode, leading towards the increase in TWR. However, a further rise in discharge current magnitude lowers the wear of the electrode because of the breakdown of dielectric molecules [55]. This acts as a source of carbon particles, which adhered to the tool surface. Eventually, tool wear is compromised.

The comparison of the TWR achieved against the two different dielectrics revealed that the rate of tool wear is significantly lower with a graphene-based dielectric if an 8 A current is used in contest to that found with kerosene.

#### 3.2.2. Electrode

**Figure 16:**
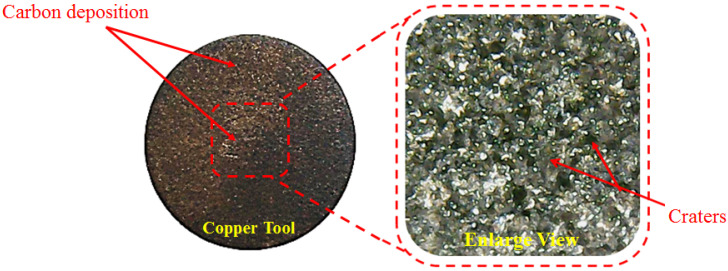
Micro-surface of copper tool showing carbon deposited on the surface.

The influence of the electrode material for the set response is shown in the plot presented in Figure 14b. It is deducted from the line plot that all the tool materials give different values of TWR. Interestingly, both types of dielectrics demonstrate a similar trend with regard to the tool materials. Such as, the value of TWR is lesser for aluminium and copper electrodes, as compared to the brass electrode in both the cutting scenarios, i.e., when a graphene-based dielectric is employed, whereas in the other kerosene is used. The primary reason for a higher value of the TWR of the brass electrode is its low thermal conductivity (109.0 W/m.K). The smaller conductivity of the electrode hinders the conduction of the heat flux in the body of the tool and thus heat is retained at the interface of the tool. This causes the melting of the electrode, which translates to higher TWR. However, the maximum TWR of brass in the case of the graphene-based dielectric is lower, as compared to the TWR of brass in kerosene. It is noticed that the copper electrode has the lowest wear value amongst the available alternatives. The better thermal conductivity of copper is accountable for this reduction. Another factor that contributes to the low TWR of copper is the formation of a redeposited layer of carbon that hampers the melting of the electrode, as evidenced in Figure 16. Overall, the TWR is smaller in magnitude if graphene nanoparticles are mixed in the dielectric. The literature revealed that the presence of powder in the cutting regime provides lower roughness and TWR [56]. This is attributed to the fact that the inclusion/mixing of the powder or nanoparticles hinder the ions to strike the tool surface. Moreover, their presence also lessens the energy of ions during collision with the electrode. Hence, it is stated that the use of a graphene-mixed dielectric yields lower wear of the tool due to spark stabilization and enhanced hindrance in the strike of the ions with the electrode.

The one-to-one comparison of the electrode materials in terms of TWR was also performed and is highlighted in Figure 14b. Based on the comparison, it can be concluded that graphene addition in the dielectric has a positive impact on the TWR of all three electrodes.

#### 3.2.3. Polarity

It is noticed that the magnitude of TWR is different at positive and negative tool polarities in the EDM of the selected workpiece material, as demonstrated in Figure 14c. It has been noticed that, at positive tool polarity, the TWR is of a smaller value rather than the negative polarity of the tool, despite whichever dielectric is used [57]. For graphene-based dielectric, the tool wear rate is slightly higher than the kerosene-based dielectric liquid at the positive polarity of the tool. The deposition of carbon particles at the tool surface in the case of the kerosene dielectric is the main reason behind this trend. On the contrary, the existence of nano particles in the kerosene lowers the chance of such a deposition and the tool surface is bare to be exposed to the penetrating heat flux. This harsh environment causes the melting of the tool surface and, eventually, TWR is aggravated. The severity of the wearing of the tool is found to be more pronounced when the electrode bears the negative polarity. The TWR observed at said polarity is lower if the graphene is mixed in the dielectric instead of using the kerosene only. Hence, the lower wear rate of tool is achieved when powder mixed dielectric is used in comparison to kerosene liquid at negative polarity.

In a nut shell, the positive polarity of the tool is appreciated in minimizing the wear of the tool. This polarity of the tool ensures the excessive heat dissipation in the surface of the workpiece at the end of the discharge duration and helps in the stabilisation the spark. Ultimately, TWR is decreased.

#### 3.2.4. Pulse–Time Ratio

The effect of the pulse–time ratio (ratio between pulse on-time to pulse off-time) on tool wear rate is illustrated in the Figure 14d. The magnitude of TWR decreases with the increase in the value of pulse–time ratio from 0.5 to 1.0. This is due to the carbon layer that is gathered on the electrode surface. The deposited layer gets thicker with the increase in T_on_ from 25 to 50 µsec while keeping T_off_ constant at 50 µsec. This carbon layer acts as a wear-resistant layer for the electrode and assists in reducing its wear. Another reason that contributes to the lowering of TWR is the expansion of the plasma channel at larger values of on-time. Such a kind of expansion of the plasma channel hinders the effective transfer of heat energy to the workpiece and the tool as well. Since a meagre heat input is experienced by the tool, thereof its wear in notably reduced. It is pertinent to mention that the trend of said control variable is similar for graphene-mixed and kerosene dielectrics in the EDM of Ti6Al4V, as depicted in Figure 14d. Further increase in the magnitude of pulse–time ratio from 1.0 to 1.5 enhanced the tool wear rate. This is credited to the reason that high pulse–time ratio means that spark discharge insists on the longer duration. This produces an excessive heat input in the work surface, as well as in the tool, which causes larger melting of the workpiece and the electrode. Consequently, TWR is increased. Hence, pulse–time ratio (1.0) is best suited for the TWR aspect during the EDM of Ti6Al4V. It is worth mentioning that the experiments performed with the graphene-mixed dielectric give more satisfying results in terms of TWR. When the graphene-mixed dielectric is compared to the kerosene-based dielectric, TWR is lower for the graphene dielectric at all levels of pulse–time ratio.

#### 3.2.5. Flushing Time

**Figure 17:**
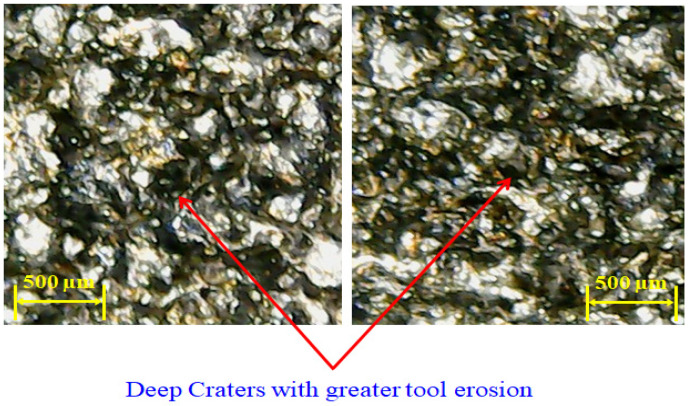
Optical micrographs showing deep craters with high tool wear rate at 8 µsec.

Flushing time is the time which is required to remove the debris between the workpiece and electrode gap. The impact of flushing time on tool wear rate is displayed in Figure 14e. It is depicted that TWR is continuously increasing with the upsurge in flushing time in the case of the graphene mixed dielectric. At 4 µsec, the TWR is lower because debris are diffused on the surface of workpiece and the electrode because of insufficient time for debris removal which led to the lowering of tool wear. On the other end at high value of flush time, eroded material is efficiently flushed away by the dielectric [58]. The dielectric also contains nanoparticles, which also act as force points for the workpiece for the electrode. The continuous circulation of this slurry imposed a kind of force to remove the melted debris from the melt pool and also took away the electrode material from the tool interface, which gets soft, owing to the exposure to the heat flux. The outcome of this is the wearing of the tool/electrode. The deterioration of the electrode surface in this regard can also been seen in the optical micrographs shown in Figure 17. The surfaces of the tools are crowded with deep craters if graphene particles are mixed in the kerosene, as witnessed in Figure 17.

The behaviour of flush time (under kerosene as dielectric) for TWR is dissimilar to that observed in the case of nano particle slurry. Initially, TWR decreases with the rise in said input parameter up to 6 µsec but, afterwards, the opposite trend is noticed. The lowering of TWR until 6 µsec is credited to the solidification of the melted droplets on the electrode surface because of the phenomenon of quenching [20]. However, as the magnitude of this variable raised beyond 6 µsec, TWR suddenly increased. This happens because of the efficient removal of debris, due to an increase in the time for the flushing of debris. Based on the aforementioned arguments, it is specified that 4 µsec of flushing time, along with the use of graphene-based slurry as a dielectric, assures the minimum level of tool wear in the EDM of Ti6Al4V.

#### 3.2.6. Spark Voltage

The influence of spark voltage on TWR using the graphene- and kerosene-based dielectric is provided in Figure 14f. It is depicted that an increase in spark voltage produces an intense electric field around the work area, which led to the localized wear of the tool and workpiece as well [8]. The melting of the electrode thereby results in more wearing of the electrode. This is the prime reason for the enhancement of the TWR when the spark voltage is shifted from 3 to 4V. At 3V, the graphene-based dielectric showed a high value of TWR because of nanoparticles, which increase the intensity of the spark but in a reasonable amount. Additionally, then TWR suddenly decreased after 4 up to 5V. This behaviour is owing to the unfavourable breakdown of the dielectric at a high spark voltage. Beside this, the enlargement of the spark gap also takes place at a high spark voltage. This minimizes the intensity of the strike of positive ions on the tool surface and led to lower TWR [59]. Moreover, the wider the spark gap, the lower the amount of heat energy that gets transmitted to the electrode, which also contributes to minimizing the TWR. It is important to note that this control parameter also portrays the similar trend for the two types of dielectrics in the EDM of Ti6Al4V.

## 4. Development and Validation of Optimal Parametric Combination

Considering the effects of all the parameters accumulatively, it is concluded that the optimal combination of parameters for achieving the maximum MRR at negative tool polarity using brass under the graphene-mixed dielectric is current = 10 A, pulse–time ratio 1, flushing time = 6 µsec and spark voltage = 4 V. On the other hand, to get a lower value of TWR, the most preferable levels of parameters are (current, electrode material, polarity, pulse–time ratio, flushing time, and spark voltage are 8 amps, copper electrode, positive polarity, 1.0 PTR, 4 µsec, and 3 V, respectively) under graphene nanoparticle slurry as the dielectric. The optimum conditions in the case of the kerosene dielectric are current (6 amps), electrode material (copper), polarity (positive), pulse–time ratio (1.0), flushing time (6 µsec), and spark voltage (3 V) as present in Table 6. The proposed settings have also been validated through confirmation trials. The results of the trials are provided in Table 6. The optimized results of optimal settings against two responses (MRR and TWR) have also been proposed and are represented in Table 7.

It is inferred that, when graphene nanoparticles are used in the kerosene dielectric, then the material removal rate is enhanced, and tool wear rate is reduced significantly. Basically, the conductivity of these particles is quite appreciable, i.e., 8 × 10^4^ S/m. Therefore, the presence of graphene nanoparticles influences the discharge characteristics. Their availability is a source of plasma channel’s expansion between tool and workpiece. Ultimately, the strength of the discharge breakdown got affected. These particles also played a pivotal role in the flushing of etched chips, thus, reducing the probability of abnormal sparking. Subsequently, higher MRR is the outcome. Furthermore, in the initial discharge phase of EDM the current magnitude gradually improved which reduced the impact of electron-to-electrode attack. Hence the material loss from the electrode surface notably reduces.

## 5. Conclusions

This research focuses on evaluating the cutting performance of the graphene-based kerosene dielectric for the two core contributing aspects (MRR and TWR) of the sustainable electric discharge machining of titanium alloy (Ti6Al4V). Experiments were performed using three different electrode materials, i.e., aluminium, brass and copper, at both positive and negative tool polarities. In total, 36 experiments were performed; 18 were with kerosene and the rest of the experiments were with the graphene-based dielectric under L18 Taguchi design. The dielectric is used with the powder-mixed graphene-based nanoparticles. The MRR and TWR have been investigated using the graphene dielectric to achieve the optimum parameters for the machining of titanium alloy. The selection of the most preferable tool material with suitable polarity was carried out in order to have maximum material removal rate and minimum tool wear rate. Based on the experimentation findings and their detailed dissuasion, along with the physical evidence, the following conclusions are drawn:

- The graphene-based dielectric outperforms MRR when compared with kerosene. The maximum value of MRR achieved with the graphene-based dielectric is 7.60 mm^3^/min, which is 64.5% higher in comparison to the maximum MRR obtained using the kerosene dielectric, i.e., 4.62 mm^3^/min.
- With respect to the tool material, the electrode of brass provides the highest value of MRR when graphene mixed dielectric was used, whereas the maximum MRR in the case of kerosene is obtained with the aluminium electrode.
- The tool wear of the Cu electrode is found to be minimal (0.17 mg/min) when graphene nanoparticles are added in the kerosene. This value of is approximately 1.5 times lower than that achieved with kerosene only.
- The magnitudes of MRR and TWR are especially sensitive to the variation in the tool polarity. The MRR and TWR are both at maximum at negative polarity. Hence, negative polarity is good for achieving a high material removal rate. However, tool wear rate for all electrode materials is lower at positive polarity, thus positive tool polarity is suitable for TWR.
- The optimal combination of parameters for achieving the maximum MRR at negative tool polarity using brass under the graphene-mixed dielectric is current = 10 A, pulse–time ratio 1, flushing time = 6 µsec and spark voltage = 4 V.
- The minimal magnitude of TWR was attained with the positive polarity using a copper electrode in graphene-based slurry while keeping the values of current, pulse–time ratio, flushing time and spark voltage at 8 A, 1, 4 µsec and 3 V, respectively.

Comparing the results obtained for graphene mixed dielectric for the kerosene, it has been revealed that the addition of the graphene nanoparticles is appreciable in achieving the sustainable EDM of Ti-alloy.

In the future, a detailed investigation will be carried out seek the impact of particles’ morphology on the cutting characteristics. Moreover, the surface integrity of the machined cavity will also be comprehensively examined.

## Figures and Tables

**Table 1:** Composition of workpiece.

Element	Ti	Al	V	O	Fe	C	H	N
% by Weight	90.0	6.42	4.22	0.19	0.15	0.06	0.003	0.0055

**Table 2:** Salient attributes of workpiece [12].

Properties	Magnitude	Units
Hardness	36	HRC
Melting Point	1604–1660	°C
Density	4.43	g/cm^3^
Elastic Modulus	113	GPa
Ultimate Tensile Strength	832	MPa
Specific heat capacity	0.5263	J/g °C
Shear Strength	550	MPa
Electrical Resistivity	1.724 × 10^−6^	ohm-m
Thermal Conductivity	6.7	W/m K

**Table 3:** Input Parameters and their values.

Control Variables	1st Level	2nd Level	3rd Level
Servo Voltage	3 V	4 V	5 V
Current	6 A	8 A	10 A
Pulse time ratio	0.5	1	1.5
Flushing Time	4 µsec	6 µsec	8 µsec
Polarity	Positive (1)	Negative (2)	--
Electrode material	Al (1)	Brass (2)	Cu (3)

**Table 4:** Characterization of graphene nanoparticles [45].

Characteristics	Magnitude/Value
Colour	Black/grey powder
Thickness	2–10 nm
Radius	1–5 µm
Density	(6–9) × 10^−2^ g/mL
Carbon percentage	>99%
Electrical conductivity	8 × 10^4^ S/m
Surplus Impurities	<1wt. %
Water Percentage (wt.)	<2wt. %
Specific surface area	20–40 m^2^/g

**Table 5:** Input parameters and output responses for graphene and kerosene-based dielectric.

Exp. No.	Input Parameters						Output Responses			
	Polarity	Electrode	Spark Voltage	Current	Pulse Time Ratio	Flushing Time	Graphene Based Dielectric		Kerosene Based Dielectric	
							MRR	TWR	MRR	TWR
1	+ve	Al	3	6	0.5	4	0.319	2.010	0.120	4.520
2	+ve	Al	4	8	1.0	6	0.493	2.410	0.243	16.14
3	+ve	Al	5	10	1.5	8	0.176	3.000	0.193	10.12
4	+ve	Brass	3	6	0.5	4	0.136	29.02	0.494	31.80
5	+ve	Brass	4	8	1.0	6	2.277	58.03	0.830	41.38
6	+ve	Brass	5	10	1.5	8	0.258	38.24	2.564	33.60
7	+ve	Cu	3	6	0.5	4	0.521	0.170	0.088	0.440
8	+ve	Cu	4	8	1.0	6	1.428	0.960	0.508	1.390
9	+ve	Cu	5	10	1.5	8	1.051	1.150	1.508	1.250
10	-ve	Al	3	6	0.5	4	0.713	25.71	1.832	38.18
11	-ve	Al	4	8	1.0	6	4.685	63.91	4.621	70.91
12	-ve	Al	5	10	1.5	8	3.312	52.88	4.119	10.21
13	-ve	Brass	3	6	0.5	4	3.405	19.33	0.648	15.32
14	-ve	Brass	4	8	1.0	6	7.602	55.20	2.254	39.56
15	-ve	Brass	5	10	1.5	8	5.615	30.00	1.266	35.40
16	-ve	Cu	3	6	0.5	4	6.616	1.200	0.548	0.490
17	-ve	Cu	4	8	1.0	6	1.131	1.620	1.209	1.800
18	-ve	Cu	5	10	1.5	8	0.343	1.940	0.760	3.920

**Table 6:** Optimal settings with their magnitudes against responses.

Responses	Optimal Setting for Graphene Mixed Dielectric	Response Magnitude with Graphene Dielectric	Optimal Setting for Kerosene Dielectric	Response Magnitude with Kerosene Dielectric	Improvement in Response Magnitude
Material removal rate (mm^3^/min)	Current: 10 Amps	7.602	Current: 6 Amps	4.621	64.5%
	Electrode: Brass		Electrode: Aluminium		
	Polarity: Negative		Polarity: Negative		
	Pulse time ratio: 1.0		Pulse time ratio: 0.5		
	Flushing time: 8 µsec		Flushing time: 4 µsec		
	Spark voltage: 4 V		Spark voltage: 5 V		
Tool wear rate (mg/min)	Current: 8 Amps	0.170	Current: 6 Amps	0.440	1.5 times
	Electrode: Copper		Electrode: Copper		
	Polarity: Positive		Polarity: Positive		
	Pulse time ratio: 1.0		Pulse time ratio: 1.0		
	Flushing time: 4 µsec		Flushing time: 6 µsec		
	Spark voltage: 3 V		Spark voltage: 3 V		

**Table 7:** Multi-object optimization of optimal setting.

Responses	Optimal Setting for Graphene Mixed Dielectric	Response Magnitude with Graphene Dielectric	Optimal Setting for Kerosene Dielectric	Response Magnitude with Kerosene Dielectric
Material removal rate (mm^3^/min)	Current: 6Amps Electrode: Copper Polarity: Negative Pulse time ratio: 0.5 Flushing time: 4 µsec Spark voltage: 3 V	6.616	Current: 10 Amps Electrode: Aluminium Polarity: Negative Pulse time ratio: 1.5 Flushing time: 8 µsec Spark voltage: 5 V	4.119
Tool wear rate (mg/min)		1.200		10.21

## Data Availability

The data presented in this study are available on request from the corresponding author.
